# Effective Synthesis of High-Integrity mRNA Using In Vitro Transcription

**DOI:** 10.3390/molecules29112461

**Published:** 2024-05-23

**Authors:** Wei He, Xinya Zhang, Yangxiaoyu Zou, Ji Li, Chong Wang, Yucai He, Qiuheng Jin, Jianren Ye

**Affiliations:** 1 College of Biology and the Environment, Nanjing Forestry University, Nanjing 210037, China; wwh09006@163.com; 2 Vazyme Biotech Co., Ltd., Nanjing 210037, China; zhangxinya@vazyme.com (X.Z.); zouyangxiaoyu@vazyme.com (Y.Z.); lijikelly@163.com (J.L.); fine1314@126.com (C.W.); 3 School of Pharmacy, Changzhou University, Changzhou 213164, China

**Keywords:** mRNA integrity, fragmented mRNA, transcription, T7 RNA polymerase, mutation, synthesis

## Abstract

mRNA vaccines are entering a period of rapid development. However, their synthesis is still plagued by challenges related to mRNA impurities and fragments (incomplete mRNA). Most impurities of mRNA products transcribed in vitro are mRNA fragments. Only full-length mRNA transcripts containing both a 5′-cap and a 3′-poly(A) structure are viable for in vivo expression. Therefore, RNA fragments are the primary product-related impurities that significantly hinder mRNA efficacy and must be effectively controlled; these species are believed to originate from either mRNA hydrolysis or premature transcriptional termination. In the manufacturing of commercial mRNA vaccines, T7 RNA polymerase-catalyzed in vitro transcription (IVT) synthesis is a well-established method for synthesizing long RNA transcripts. This study identified a pivotal domain on the T7 RNA polymerase that is associated with erroneous mRNA release. By leveraging the advantageous properties of a T7 RNA polymerase mutant and precisely optimized IVT process parameters, we successfully achieved an mRNA integrity exceeding 91%, thereby further unlocking the immense potential of mRNA therapeutics.

## 1. Introduction

Since the first mRNA vaccine (BNT162b2) was approved by the FDA at the end of 2020, mRNA-based therapies have experienced rapid development [1,2,3,4,5]. Notably, mRNA drugs have the following benefits: tremendous potency against various diseases, including infectious diseases, cancers, and rare diseases; extremely low dosage for greater security; incredibly rapid drug discovery; and large-scale drug development [6,7,8,9,10,11].

Traditional mRNA consists of a 5′cap, 5′untranslated region (UTR), open reading framework (ORF), 3′untranslated region (UTR), and a poly (A) tail [12]. mRNAs are generally synthesized via in vitro transcription (IVT) using RNA polymerases (RNAPs) [13,14,15,16]. T7 RNA polymerase (T7 RNAP), encoded by bacteriophage T7, is the most thoroughly characterized member of a widespread family of RNAPs [17,18] and has already been used to successfully synthesize the first accepted mRNA vaccine [19]. T7 RNAP transitions from an initiation phase, which involves binding to the T7 promoter and the initiation of de novo mRNA synthesis, to an elongation phase releasing the RNA product through the mono subunit [20,21]. Impurities are inevitably generated during this complex formational process. The most common impurity is the immunogenic double-stranded RNA (dsRNA) byproduct, although the rate of dsRNA production is relatively low [22]. Nevertheless, the activation of pattern recognition receptors (RIG-I, MDA5 and so on) by dsRNA has adverse consequences, including the inhibition of protein synthesis and cell death, which can impact the efficacy of mRNA therapy [23,24,25].

**Figure 1:**
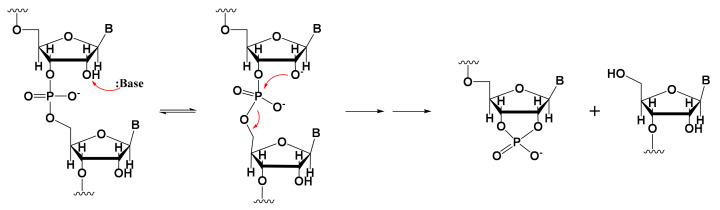
Schematic showing one theory of metal-based RNA degradation.

Fragmented mRNA is another common impurity that must be taken into account [26,27]. Only full-length mRNA transcripts containing both a 5′-cap and a 3′-poly(A) structure are viable for in vivo expression. mRNA fragmentation is often caused by mRNA degradation [28,29,30,31], in which hydrolysis plays a key role, and which can be promoted by alkaline or metal ions such as Mg^2+^ [32,33]. This metal-based catalytic mechanism of degradation can occur in two ways. First, hydrated metal magnesium ions cleave RNA as Bronsted bases and extract protons from the 2′-OH ribose group; the resulting 2′-O group undergoes nucleophilic substitution of phosphorus atoms, leading to the departure of 5′-OH. The collinear geometric structure between the nucleophilic reagents and leaving groups then promotes the degradation reaction (Figure 1). Second, metal magnesium ions can stabilize degraded RNA intermediate products (the negative ions of the leaving group) to promote the forward reaction of degradation [32].

**Figure 2:**
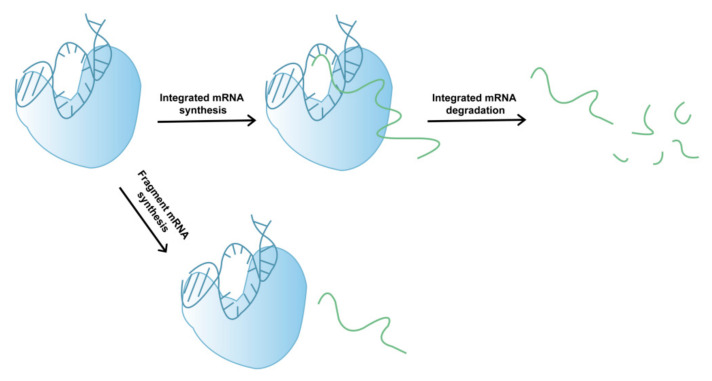
Schematic showing the generation of mRNA fragments.

In addition to its involvement in mRNA degradation, fragmented mRNA may also be produced during the complex process of T7 RNAP-catalyzed mRNA synthesis (Figure 2). During transcription, several factors can lead to early termination, whereby mRNA products leave the DNA template and fragmented mRNA is generated. The exact mechanisms of early mRNA termination during T7 RNAP-catalyzed mRNA transcription remain relatively unclear, which hinders our ability to devise rational approaches for reducing fragmented mRNA synthesis and improving mRNA integrity. However, low-quality (low-integrity) mRNA may reduce the therapeutic effect of drugs and induce other adverse reactions in later stages [34,35]. Moreover, as mRNA drugs have only recently been approved by the FDA, long-term clinical data are currently absent. Therefore, to avoid potential adverse reactions, such as those caused by dsRNA byproducts, the integrity of mRNA products must be urgently improved.

There is a lack of research on improving mRNA integrity. Research into dsRNA byproducts shows that they can be reduced through the optimization of the reaction buffer, improving the efficiency of purification or other effective methods [13,36,37], whereas the most effective method is the design of T7 RNAP [22]. Indeed, the use of the T7 RNAP variants G47A + 884G as a catalyst for mRNA IVT can effectively decrease dsRNA generation and simplify downstream purification processes for dsRNA removal. Similarly, the production of mRNA fragments may also be closely related to T7 RNAP. Thus, T7 RNAP engineering shows potential for reducing mRNA impurities and improving mRNA integrity.

**Figure 3:**
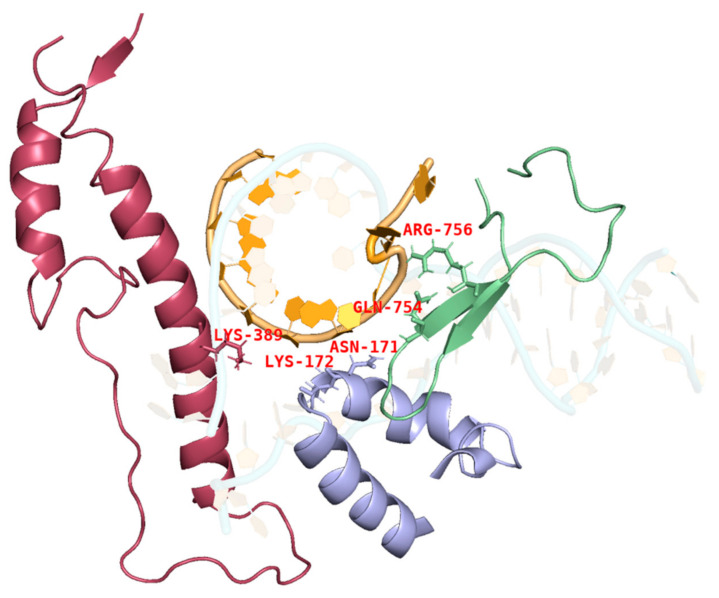
Interactions among the site chains of amino acids K389, K172, N171, Q754, and R756 from subdomain H (purple), the specificity loop (green), and the thumb domain (red) with mRNA products (orange).

During IVT, the DNA template, RNA products, and T7 RNAP form a complex that ensures the smooth progression of nucleotide addition cycles to extend the mRNA chain along the DNA template. The thumb domain of T7 RNAP maintains the stability of the transcription complex [38,39,40]. T7 RNAP K389, which forms the helix-N of the thumb domain, is an important structure influencing the complex stability, whereas N171, K172, N754, and R756, which form the subdomain H and specificity loop within the hydrogen-bonding distance of phosphates at the 5′end of the single-stranded mRNA, may accelerate mRNA separation from the DNA template to ensure better formation of longer RNA (Figure 3) [41,42,43]. It is likely that accelerated separation of the DNA template and RNA products causes unstable binding between the RNA products and DNA and leads to the early termination of the transcription. Thus, RNA products leave the complex in advance of mRNA fragmentation.

In this study, we explore the possibility of synthesizing higher-integrity mRNA through IVT. Specifically, we focus on using T7 RNAP substitutions, as well as an optimized DNA template, to reduce the proportion of fragmented mRNA products. This research improves our understanding of factors influencing mRNA integrity and provides a basis for developing more efficient mRNA-based therapies with fewer impurities.

## 2. Results and Discussion

### 2.1. Fragmented mRNA Produced In Vitro Transcription

**Figure 4:**
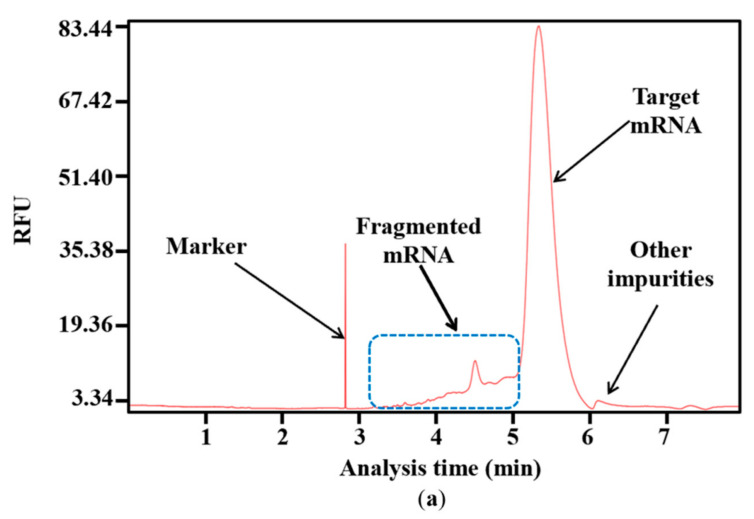

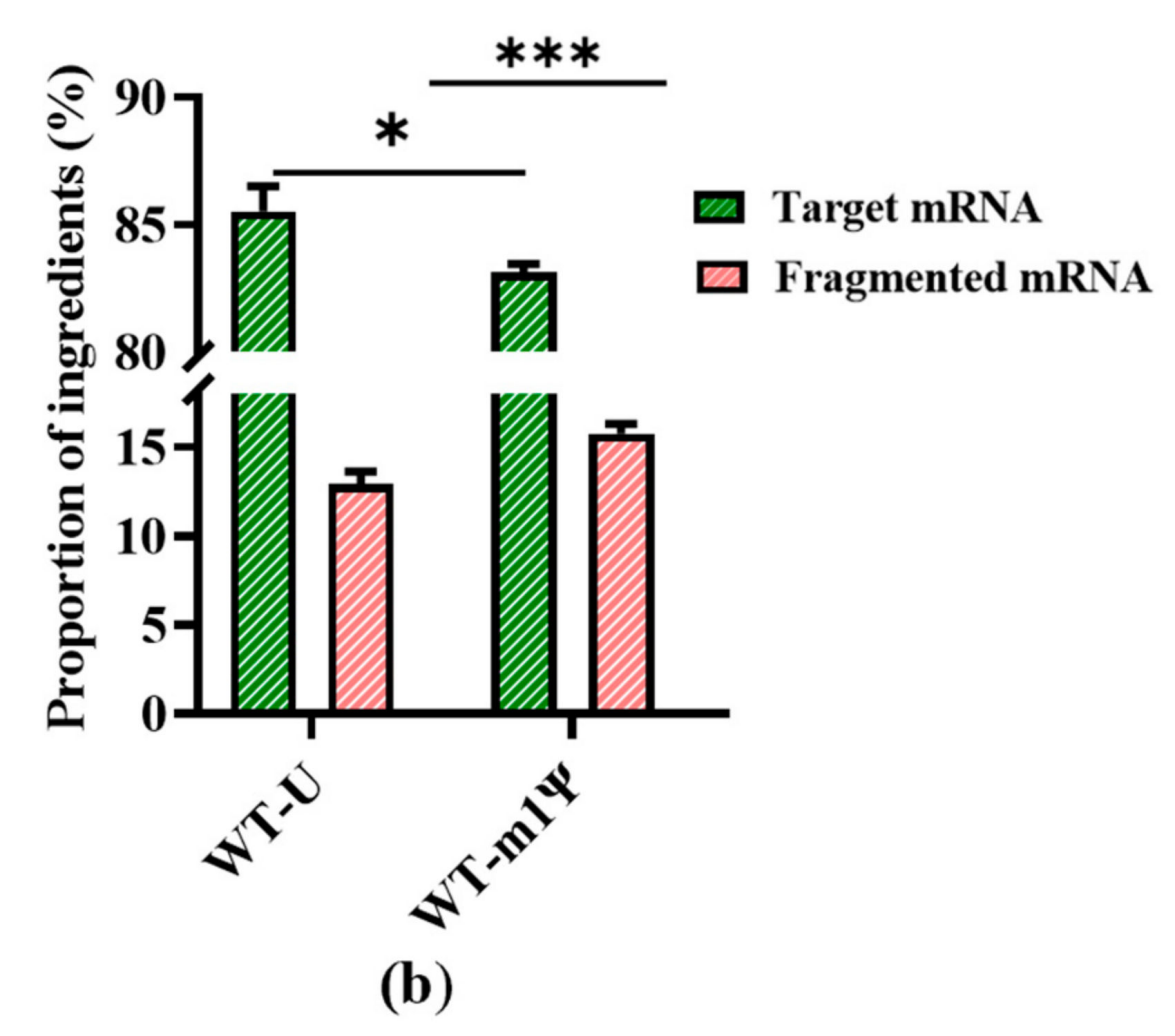
Analysis of mRNA products: (**a**) capillary electrophoresis analysis; (**b**) comparison of mRNA integrity and proportion of fragmented mRNA generated when using uridine (U) and m1ψ as substrates (the experiments were replicated ≥4 times; error bars represent ±SD; * *p* < 0.05, *** *p* < 0.001).

Impurities are inevitably generated during the complex process of RNA synthesis [44]. Based on the results of capillary electrophoresis, which is the classical method of distinguishing the molecular weight of RNA products [45], the proportion of target mRNA among the mRNA products was only 85.53 ± 0.99%, and nearly 15% of the mRNA products were impurities. Of these, 89.39 ± 2.84% of the impurities were fragmented mRNA products, while nearly 11% were other impurities with a molecular weight greater than the target mRNA (Figure 4a,b). When determined according to mass, fragmented products are clearly visible, as indicated in Supplementary Figure S2. This large proportion of fragmented mRNA products has the potential to induce other side reactions that may affect treatment effectiveness. Moreover, the substantial generation of fragmented mRNA results in wasted raw materials and complex downstream purification. Thus, reducing the amount of fragmented mRNA is a priority.

In previous research, the generation of dsRNA is problematic because of the severe immunogenic reaction that occurs after injection into the body. However, the use of the modified nucleic acid m1ψ can significantly reduce dsRNA production. Due to this creative innovation, Karikó and her colleague won the 2023 Nobel Prize in Physiology or Medicine [46,47,48,49]. mRNA vaccines accepted by the FDA replace uridine with m1ψTP as the raw material to limit the potential for immunogenicity [50,51,52].

However, our results revealed a new issue. When was uridine replaced by m1ψ as the raw material, the yield of mRNA products was similar (Table S1, in Supplementary Material), while the proportion of fragmented mRNA increased. As shown in Figure 4b, the proportion of fragmented mRNA was 15.78 ± 0.52%; thus, the integrity of mRNA products decreased to 83.13 ± 0.34%. (the experiment was then replicated 20 times, Figure S3, in Supplementary Material). This may be attributed to a change in the interaction with the mRNA secondary structure, which resulted in an unstable transcription complex (DNA template–T7 RNAP–mRNA products). This change may have further facilitated the early separation of mRNA from the DNA template and its subsequent fragmentation. This increased mRNA fragmentation highlights the need for an efficient strategy to reduce impurities and simplify the downstream purification steps.

### 2.2. T7 RNAP Substitution Screening for Reducing Early Termination and the Formation of Fragmented mRNA

**Figure 5:**
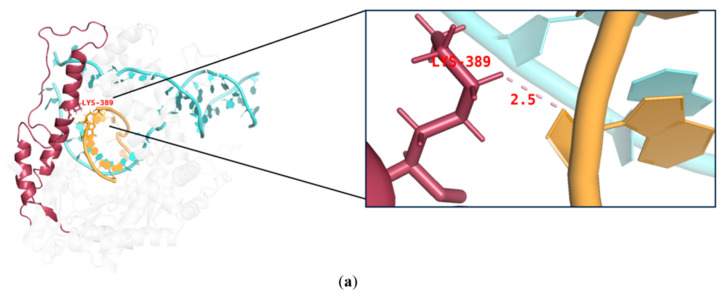

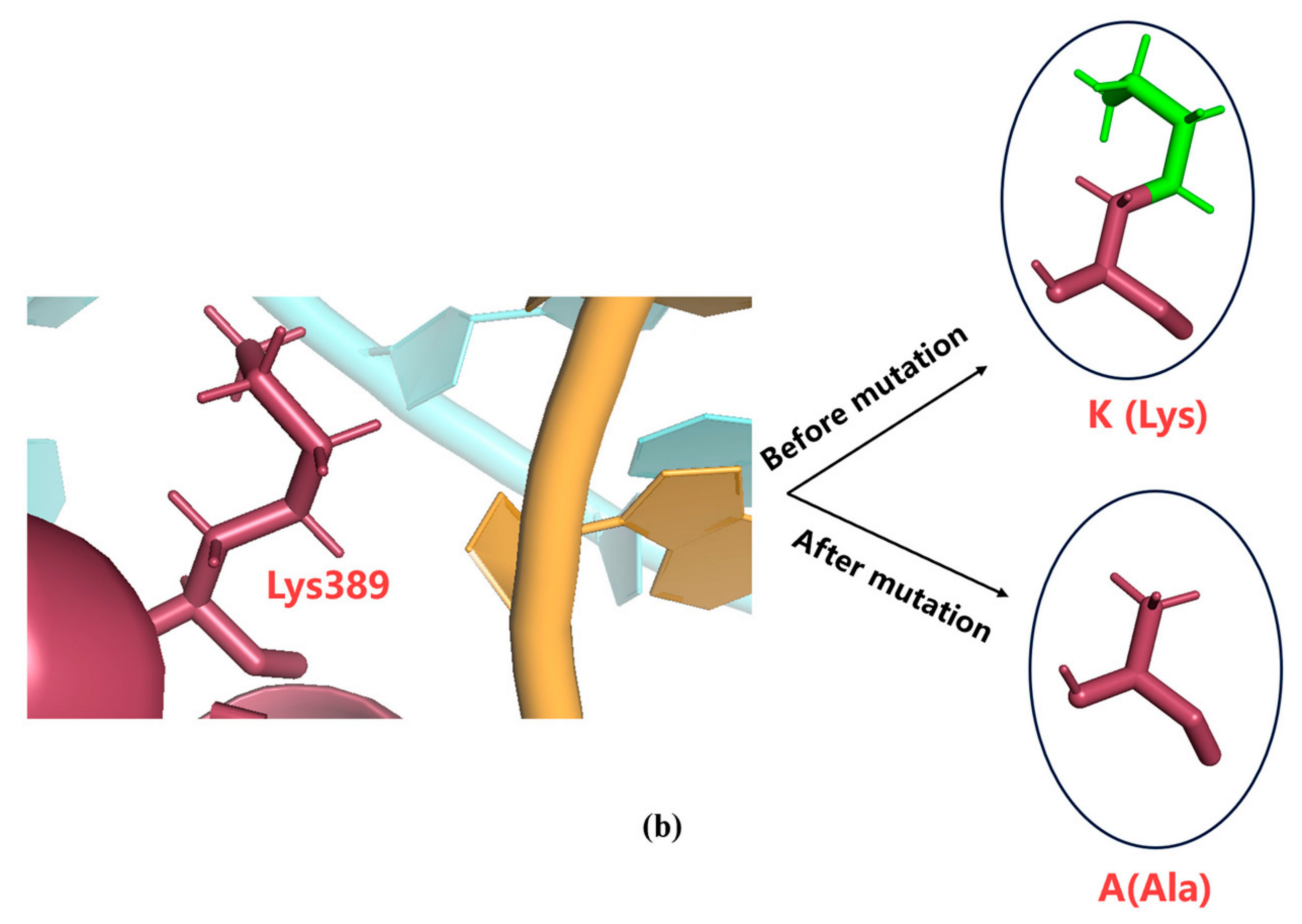
Structural analysis of (**a**) T7 RNAP WT and (**b**) T7RNAP K389A.

mRNA fragmentation can be generated by a pause in transcription. According to previous research, T7 RNAP K389, N171, K172, N754, and R756 found within hydrogen-bonding distance of phosphates at the 5′end of single-stranded mRNA may accelerate the separation process of mRNA from the DNA template, enabling better formation of longer RNA [41]. This earlier separation may lead to a pause in transcription, generating fragmented mRNA. In this study, we used alanine instead of the original amino acids. The side chain of alanine is -CH_3_ (methyl); this greatly extended the distance between amino acids and nucleic acids (see K389A as an example in Figure 5). Furthermore, H from -CH_3_ cannot easily form hydrogen bonds.

**Figure 6:**
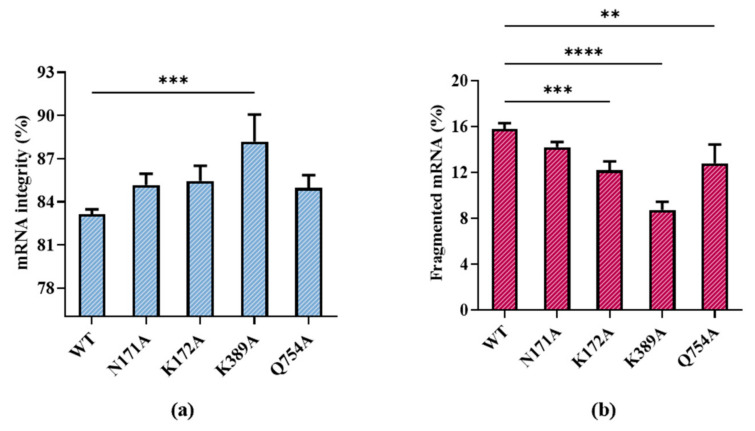
Effect of T7 RNAP substitutions on (**a**) mRNA integrity (%) and (**b**) fragmented mRNA (%) (the experiments were replicated ≥4 times; error bars represent ±SD; ** *p * <  0.01, *** *p*  <  0.001, **** *p*  <  0.0001).

According to our results, all substitutions except for R756A showed improved integrity. R756A is from a specific loop, the crucial function functional structure for T7 promoter recognition. R756A may have induced the failure of mRNA transcription by losing the ability to identify the T7 promoter [41]. Among the remaining substitutions, T7 RNAP K389A improved mRNA integrity, which resulted in the proportion of target mRNA becoming 88.18 ± 1.89%, while the proportion of fragmented mRNA decreased to 8.73 ± 0.72% (Figure 6a,b).

The integrity of products may be related to various factors such as the integrity of the DNA template, enzyme stability, reaction system, and reaction conditions. Moreover, the molecular weight of T7 RNAP is relatively large (883 aa), and it is difficult to improve the integrity using only a single site mutation. A K389 unit point mutation improved the integrity significantly, indicating that site K389 is strongly related to the integrity of RNA products. By contrast, the mutants N171A, K172A, and Q754A from different domains did not have the same magnitude of impact on mRNA integrity. Similar to the lack of effect of R756A, the correlation with integrity is not only determined by hydrogen bonds but also by the function of the domains of these sites.

### 2.3. mRNA Integrity Correlated with Amino Acid α-Helix Propensity of Site K389

**Figure 7:**
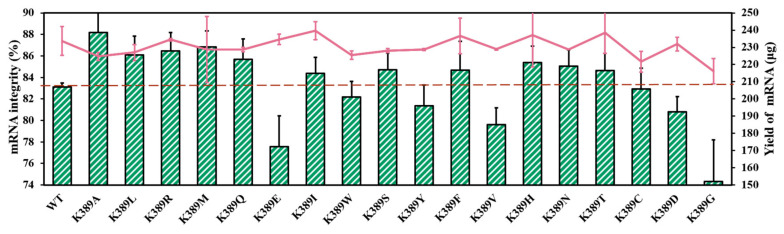
Comparison of mRNA integrity (%) (histograms) and yield of mRNA products (lines) catalyzed by various substitutions (the experiments were replicated ≥4 times; error bars represent ±SD).

To further analyze the correlation between site K389 and mRNA integrity, we performed directed site saturation testing. As shown in Figure 7 and Table S2, there are no significant distinctions in mRNA yield between T7 RNAP and substitutions [53]. It appears that the α-helix tendency of K389 is related to mRNA integrity (Figure 7 and Figure S4a,b, in Supplementary Material). The helix propensity of the amino acid Lys (K) was 0.26 kcal/mol. The replacements of Ala (0 kcal/mol), Leu (0.21 kcal/mol), Arg (0.21 kcal/mol), and Met (0.24 kcal/mol), which exhibited greater α-helix tendency than Lys, were found to have the ability to improve mRNA integrity (Figure 7). In detail, the helix tendency of these amino acids decreased in the following order: Ala > Leu ≥ Arg > Met > Lys > Gln > Glu > Ile > Trp > Ser > Tyr > Phe > Val > His > Asn > Thr > Cys > Asp > Gly (helix propensity 0–1 kcal/mol, Table S2, in Supplementary Material). Among these substitutions, T7 RNAP K389A exhibited the greatest improvement in mRNA integrity. By coincidence, the amino acid alanine exhibited the highest helix tendency.

Furthermore, with the use of the amino acid Gly (1 kcal/mol), which had the lowest α-helix tendency, the mRNA integrity decreased significantly to 74.32 ± 3.88%. When Asp (0.69 kcal/mol) and Cys (0.68 kcal/mol) were used as replacements, the synthetized target mRNA decreased to 80.82 ± 1.39% and 82.91 ± 1.94%, respectively. The substitutions in the middle values of helix propensities were not found to be dominated by the mechanism (Figure 7).

Site K389 originates from a crucial α-helix and belongs to a thumb domain that plays an important role in maintaining transcription complex stability [40]. Therefore, our results indicate that replacements with α-helix tendency at site K389 have the ability to catalyze high-integrity mRNA. Furthermore, the α-helix tendency of the site that improved mRNA integrity may be attributed to the stable α-helix structure of the original site, suggesting that the stability of the special α-helix is important for ensuring smooth nucleotide addition cycles and the synthesis of complete mRNA products.

### 2.4. Optimized DNA Template Further Increase mRNA Integrity

Here, we attempted to further improve mRNA integrity on the basis of existing improvement using T7 RNAP K389A as the catalyst. As well as the T7 RNAP catalyst, the DNA template sequence and transcribed mRNA sequence were also closely related to mRNA integrity [54,55]. In particular, rare codons have the possibility of causing premature transcription termination and abolishment of full-length mRNA in vivo and in vitro [56,57]. DNA sequence with more rare codons have higher AU contents and potentially higher chance of forming termination hairpin to trigger early termination [56,58]. For example, when a special sequence (Terminator I (rrnBT1), an AU rich sequence) that was reported to cause the formation of a termination hairpin (SEQ NO.3, in Supplementary Material) was inserted, as data from Supplementary Figure S5 show, nearly half of mRNA products were generated as fragmented mRNA.

**Figure 8:**
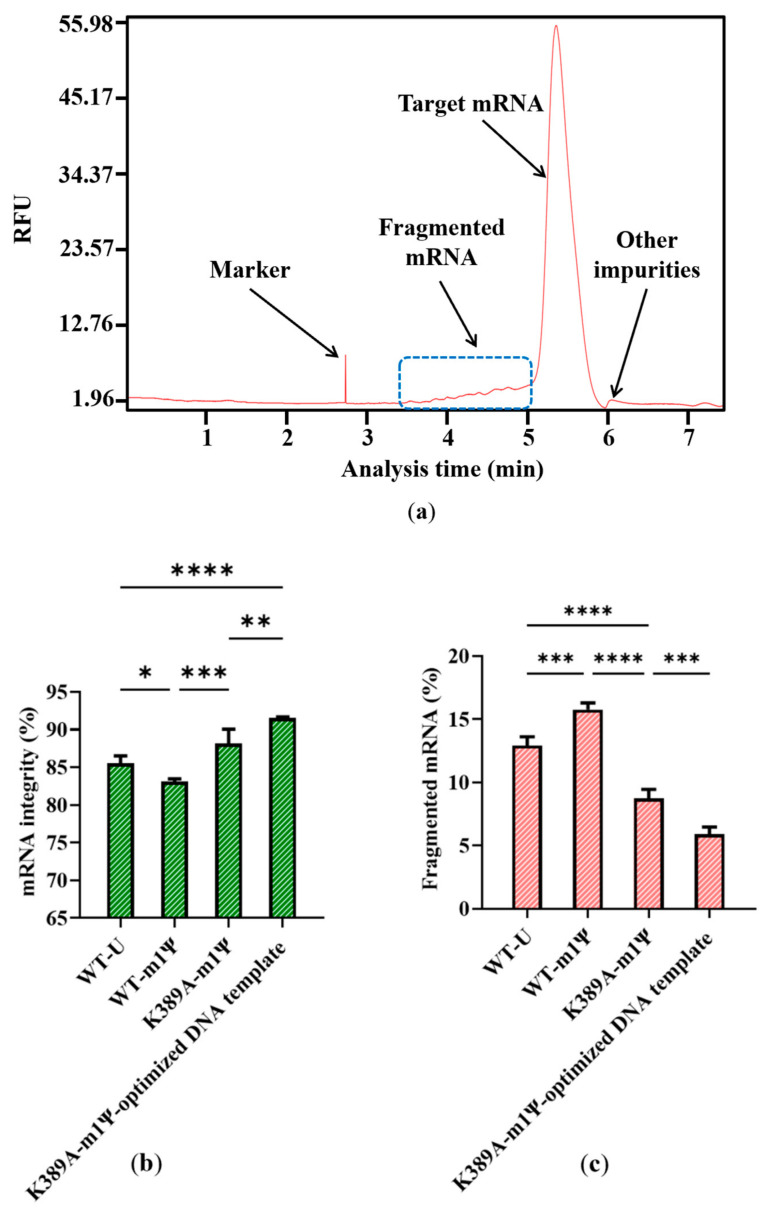
Effect of optimized DNA template on (**a**) capillary electrophoresis analysis of mRNA products; (**b**) mRNA integrity; and (**c**) proportion of fragmented mRNA (The experiments were replicated ≥4 times; error bars represent ±SD; * *p*  <  0.05, ** *p * <  0.01, *** *p*  <  0.001, **** *p*  <  0.0001).

The DNA template we used above translated the same protein as Moderna’s mRNA vaccine mRNA-1273 but was not optimized yet (SEQ NO.4, in Supplementary Material) [51,59]. We replaced the sequence with the optimized transcribed sequence of Moderna’s mRNA-1273 DNA to analyze the template’s impact on mRNA integrity (SEQ NO.5, in Supplementary Material). As expected, the integrity of mRNA products was further increased to 91.55 ± 0.13%, whereas the proportion of fragmented mRNA reduced to 5.90 ± 0.56% (Figure 8b,c).

### 2.5. Increase in mRNA Integrity via Addition of Organic Acid

**Figure 9:**
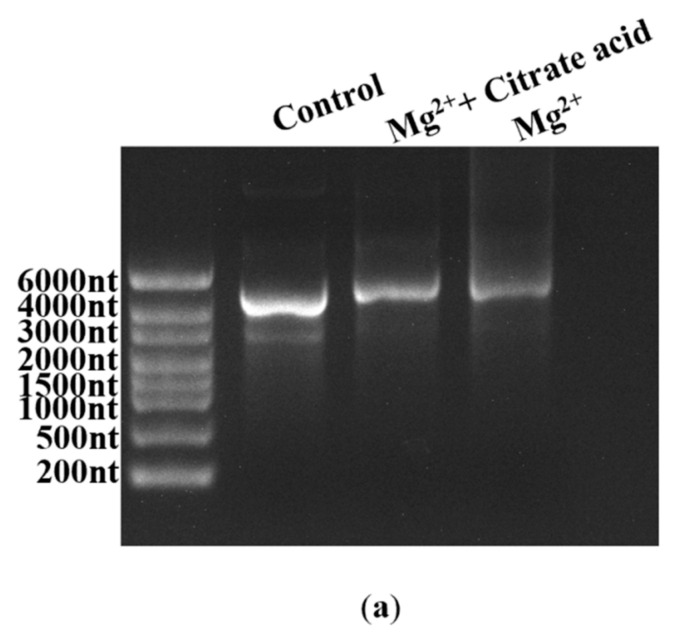

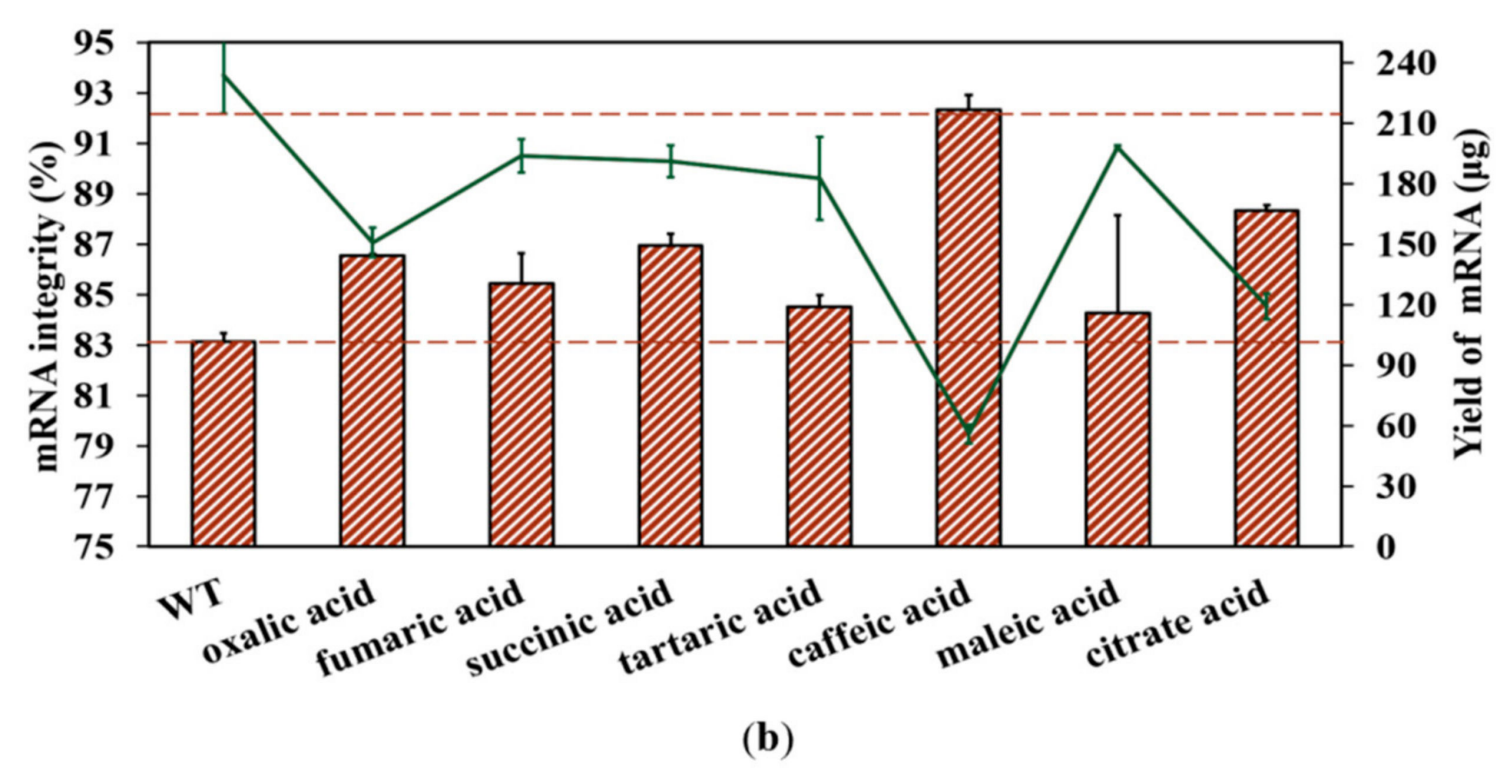
Effect of different organic acids on mRNA degradation: (**a**) citrate reduced the Mg^2+^-based degradation efficiency of mRNA; (**b**) mRNA integrity (%) (histograms) and the yield of mRNA products (lines) (the experiments were replicated ≥3 times; error bars represent ±SD).

Fragmented products are also generated by the inherent instability of the mRNA structure commonly following hydrolysis, especially crucial cofactor Mg^2+^-induced hydrolysis [32]. As shown in Figure S6 (in Supplementary Material), mRNA degradation increased as the Mg^2+^ concentration increased; in particular, the addition of 12 mM Mg^2+^ markedly enhanced the degradation reaction, coinciding well with the reported results [32,60]. While there is no effective method that has been confirmed to reduce degradation efficiency at present, Benyamin et al. found that the addition of organic acids decreased the precipitation of mRNA products caused by Mg^2+^ [61]. In this study, we also employed organic acids in an attempt to decrease mRNA fragmentation by limiting Mg^2+^-induced degradation. Firstly, an organic acid, citrate acid, was used to verify our conjecture, as shown in Figure 9a. As expected, the degradation of mRNA products was clearly reduced.

Subsequently, we added 5 mM of organic acids (oxalic acid, succinic acid, tartaric acid, caffeic acid, citric acid, fumaric acid, and maleic acid) into the reaction system. As shown in Figure 9b and Figure S7 (in Supplementary Material), all organic acids improved mRNA integrity, especially caffeic acid and citric acid. Conversely, the addition of organic acids may also impact the transcriptional active center, especially when adding caffeic acid, which results in a yield less than 50% of the original yield. This may be caused by the chelating force influencing the key mechanism of Mg^2+^ as the cofactor for mRNA IVT. As organic acids exhibit potential for reducing degradation-induced fragmentation during mRNA synthesis, further research is required to effectively chelate magnesium ions, reduce degradation efficiency, and reduce the negative impact of magnesium on the yield of mRNA products. Once this is achieved, mRNA integrity will likely be much higher when appropriate organic acids are added to the reaction system catalyzed by the T7 RNAP mutant K389A. The development of mutant T7 RNA polymerase is a hot issue, and research is focused on improving the integrity of mRNA by reducing the short-truncated RNA sequences generated by in vitro transcription [22]. Except for further improving mRNA integrity, fidelity of the replacement is also an important issue for the development of applications based on mRNA [62,63]. This analysis is out of scope of this research, but it will be studied in the near future.

## 3. Materials and Methods

### 3.1. Reagents and Chemicals

A site-directed mutagenesis kit; dsRNA quantitative kit; murine RNase inhibitor; pyrophosphatase; XBaI; and the substrates ATP, GTP, CTP, and UTP/m1ψTP were supplied by Vazyme Co., Ltd. (Nanjing, China). MgCl_2_, glycerol, Spermidine, dithiothreitol (DTT), trichloroethyl phosphate (TCEP), and other chemicals were purchased from Aladdin Industrial Inc. (Shanghai, China) and other commercial sources.

### 3.2. Cloning and Heterologous Expression of T7 RNAP

The encoding nucleotide sequence of T7 RNAP (inserting His-tag sequence at the 5′terminol of foreign nucleotide sequence) was optimized using Gene Optimizer^®^ expert software (GeneArt Services Dashboard (thermofisher.cn) for improved expression in *E. coli* (Supplementary Sequence No. 1). The optimized nucleotide sequence was amplified via PCR by the forward primer 5′-CGCGGATCCATGAATACAATTAACATAGCTAAAAATGA-3′ and reverse primer 5′-CCCAAGCTTTTACGCGAAGGCGAAATCAGATT-3′, which incorporated the BamHI and HindIII restriction sites, respectively. The sequence was then digested with BamHI and HindIII, and the insert was cloned into the plasmid pQE-30 (Novagen Inc., Madison, WI, USA) and digested with the same restriction enzymes (Figure S1a, in Supplementary Material). The recombinant plasmid pQE-30 was transformed into *Escherichia coli* BL21 (DE3) (Novagen Inc., (Madison, WI, USA)). Overnight precultures were diluted in a Luria–Bertani agar plate containing 100 mM ampicillin. The cells were induced by 0.5 mM IPTG for 4–6 h when optical density at 600 nm reached 0.6~0.8. Subsequently, the cells were harvested via centrifugation (4 °C, 10,000× *g*, 20 min).

Thereafter, cells were washed twice and resuspended in Tris-HCl buffer (pH 8.0, 50 mM), then sonicated (260 W, 3 s pulse, 5 s pause). The crude enzyme was obtained via centrifugation (4 °C, 10,000× *g*, 20 min). The protein content was analyzed using SDS-PAGE (Figure S1b, in Supplementary Material).

### 3.3. Purification of T7 RNAP

Pretreatment involved the addition of 0.1 vol of 3% polyethyleneimine to remove the impurities of nucleic acids from the crude enzyme. The sample was diluted using the same volume of sulfopropyl (SP)-binding buffer (20 mM MES, 100 mM NaCl, 5% *v*/*v* glycerol, 1 mM dithiothreitol, pH = 6.5) and then loaded onto a 1 mL SP column equilibrated with 10 mL SP binding buffer. The sample was eluted with 5 mL of SP elution buffer (40 mM MES, 500 mM NaCl, 5% *v*/*v* glycerol, 1 mM DTT, pH = 7.5). Then, T7 RNAP was diluted using the same volume of Ni-binding buffer (300 mM NaCl, 0.2 mM DTT, 20 mM imidazole, 5% *v*/*v* glycerol, 20 mM Tris-HCl pH 7.5) and loaded onto a His trap HP (GE Healthcare Corp., Chicago, IL, USA). The purified T7 RNAP was eluted with 5 mL of Ni-elution buffer (200 mM NaCl, 0.2 mM DTT, 450 mM imidazole, 5% *v*/*v* glycerol, 20 mM Tris-HCl pH 7.5). The purified T7 RNAP was desalted using a HiTrap Desalting Column (GE Healthcare Corp., Chicago, IL, USA). The sample content was measured with a BCA Protein Assay Kit (Vazyme Co., Ltd., Nanjing, China) with bovine serum albumin as the standard.

### 3.4. Measurement of Specific Activity

First, the activities of the samples (T7 RNAP wild-type (WT) and mutants) were estimated as X U/μL. The T7 RNAP standard (Vazyme Co., Ltd., Nanjing, China) and samples were gradient-diluted to 0 U/μL, 0.05 U/μL, 0.1 U/μL, 0.2 U/μL, 0.3 U/μL, and 0.6 U/μL using Tris-HCl buffer (50 mM pH 8.0) containing 100 mM NaCl, 0.1 mM EDTA-2Na, and 10% *v*/*v* glycerol. The reaction mixture (final concentrations of 0.5 mM ATP/GTP/CTP/UTP, 2 U/μL Murine RNase Inhibitor, 4 ng/μL linear DNA template (plasmid containing a target sequence digested with BspQI) (target sequence, Supplementary SEQ NO.4), 20% *v*/*v* T7 RNAP, 10 mM MgCl_2_, 2 mM Spermidine, and 40 mM Tris-HCl pH 8.0) was added to a 96-well plate so that the total volume of the reaction medium was up to 20 μL. After being incubated for 30 min at 37 °C, nucleic acid dye BR reagent (Vazyme Co., Ltd., Nanjing, China) was added. Fluorescence detection was performed at an excitation wavelength of 630 nm and an emission wavelength of 680 nm; the gain value was 126. According to the linear regression equation of the microplate reader (slope = k), the specific activity was defined as follows:

Y (Standard) = k1X + b(1)

Y (Sample) = k2X + b(2)

Activity of sample = k1/k2 × Estimated value of activity (U/μL)(3)

Specific activity of sample = Activity of protein (U/μL)/Concentration of protein (μg/μL)(4)

### 3.5. In Vitro Transcription and Measurement of mRNA Integrity

The reaction mixture for mRNA synthesis (final concentrations of 10 mM ATP, GTP, CTP, UTP/ m1ψTP, 2 U/μL murine RNase inhibitor, 0.005 U/μL pyrophosphatase, 50 ng/μL linear DNA template (Supplementary Sequence No. 3–5), 15 U/μL T7 RNAP, 30 mM MgCl_2_, 2 mM Spermidine, 2.5 mM Tcep, 40 mM Tris-HCl pH8.0) was added to a 96-well plate so that the total volume of the reaction medium was up to 20 μL. After incubation for 60 min at 37 °C, mRNA products were purified using RNA Clean Magnetic Beads (Vazyme Co., Ltd., Nanjing, China), and the mRNA yield was measured using a Nanodrop 2000C spectrophotometer. After denaturing the mRNA products (incubated for 5 min at 70 °C, then iced for 5 min at 0 °C), the integrity of mRNA products was measured using capillary electrophoresis.

### 3.6. Mg^2+^-Induced mRNA Degradation

The 5 μg RNA products; final concentrations of 0, 1, 2, 4, 8, and 12 mM MgCl_2_; and 2 U/μL of murine RNase inhibitor were added to the reaction medium, ensuring that the total volume was 20 μL. After incubation for 2 h at 37 °C, the hydrolysate was analyzed using agarose gel electrophoresis in the Gel Imaging System. Subsequently, 6 mM citrate was also added to the reaction medium with 12 mM MgCl_2_. The solution was incubated for the same duration, and the differences in hydrolysis efficiency were analyzed using agarose gel electrophoresis in the Gel Imaging System.

### 3.7. Optimization of mRNA Integrity

To optimize mRNA integrity, a final concentration of 5 mM of organic acid (oxalic acid, succinic acid, tartaric acid, caffeic acid, fumaric acid, maleic acid, and citrate acid) was mixed in the reaction medium, respectively. After incubation for 60 min at 37 °C, the mRNA products were purified, and the integrity was measured using capillary electrophoresis.

### 3.8. Fragmented mRNA Measurement Using LC-MS

The PCR-amplified DNA template was purified using DNA clean beads (Vazyme Co., Ltd., Nanjing, China), and then the yield of the DNA template was measured with a Nanodrop 2000C spectrophotometer (Supplementary Sequence No. 6). A final concentration of 50 ng/μL of PCR-amplified DNA template; 10 mM ATP, GTP, CTP, and UTP; 2 U/μL murine RNase inhibitor; 0.005 U/μL pyrophosphatase; 15 U/μL T7 RNAP; Tris-HCl 40 mM; MgCl_2_ 30 mM; Spermidine 2 mM; and Tcep 2.5 mM were added to a 96-well plate and incubated for 60 min at 37 °C. Subsequently, the RNA products were digested using DNase I and then purified by RNA using clean magnetic beads (Vazyme Co., Ltd., Nanjing, China). The yield of the RNA products was measured using a Nanodrop 2000C spectrophotometer. The proportion of impurities in the RNA products was determined with liquid chromatography–mass spectrometry (ChromCore C18 column (1.8 μm, 4.6 × 50 mm). Two solvents were used for gradient elution: 0.065% hexafluoroisopropanol 0.0375% *N*,*N*-Diisopropylethylamine dissolved in ddH_2_O (A) and 0.065% hexafluoroisopropanol--0.0375% N,N-Diisopropylethylamine dissolved in methanol (B), with a flow rate of 0.3 mL/min. The analytes were detected in negative ion mode with a full scan and a scanning range of 600–3000.

## 4. Conclusions

In conclusion, the generation of fragmented mRNA is a critical issue, especially when using m1ψ as a replacement for mRNA synthesis, which generated only 83.13 ± 0.34% complete mRNA products and a large amount of fragmented mRNA (almost 15% of all mRNA products). Such poor mRNA integrity will likely lead to decreased therapeutic efficacy and downstream purification challenges. The factors influencing mRNA fragmentation include degradation, early termination of transcription catalyzed by T7 RNAP, and sequence-specific effects. In this study, we focused on using mutants of T7 RNAP to catalyze higher-integrity mRNA products. We first identified that the reduced possibility of mutants forming hydrogen bonds with mRNA products led to improved mRNA integrity. Furthermore, mutants replaced in the T7 RNAP site K389 with amino acids exhibiting the highest α-helix propensity improved the mRNA integrity to 88.18 ± 1.89% and reduced the proportion of fragmented mRNA to 8.72 ± 0.72%. Additionally, the use of an optimized DNA template further improved the mRNA integrity to 91.55 ± 0.13% and reduced the proportion of fragmented mRNA to less than 6%. Overall, this research elucidates the factors influencing mRNA integrity and provides a basis for developing more efficient mRNA-based therapies with fewer impurities.

## Data Availability

The data presented in this study are available in article and Supplementary Materials.

## Supplementary Materials

The following supporting information can be downloaded at https://www.mdpi.com/article/10.3390/molecules29112461/s1, Figure S1: Map of recombinant plasmid pQE-30 and SDS-PAGE analysis of T7 WT; Figure S2: Mass spectra of WT-catalyzed mRNA production; Figure S3: Capillary electrophoresis analysis of mRNA products via using UTP or m1ψTP as substrate; Figure S4: (a) Relative mRNA integrity of higher helix tendency of substitutions; (b) Relative mRNA integrity of lower helix tendency of substitutions; Figure S5: Effect of DNA template sequence insertion of Terminator I (rrnBT1) on capillary electrophoresis analysis of mRNA products;. Figure S6: Mg2+-induced degradation of mRNA; Figure S7: Acids improved mRNA integrity; Table S1: Optimized DNA template and T7 RNAP K389A improved mRNA integrity; Table S2: mRNA integrity catalyzed via T7 RNAP substitutions. SEQ NO.1: Optimized cDNA sequence of T7 RNA WT; SEQ NO.2: Amino acid sequence of T7 RNAP WT; SEQ NO.3: DNA template sequence inserting Terminator I (rrnBT1) sequence; SEQ NO.4: Original DNA template sequence; SEQ NO.5: Optimized DNA template sequence of Moderna mRNA-1273 vaccine; SEQ NO.6: 30nt.
