# Supplementary material for: Effective Synthesis of High-Integrity mRNA Using In Vitro Transcription
Source: Molecules. 2024 May 23;29(11):2461. doi: 10.3390/molecules29112461 (PMC11173937; doi:10.3390/molecules29112461)
Supplement: Supplementary file 1 [file molecules-29-02461-s001.zip › molecules-2982008-supplementary.pdf]

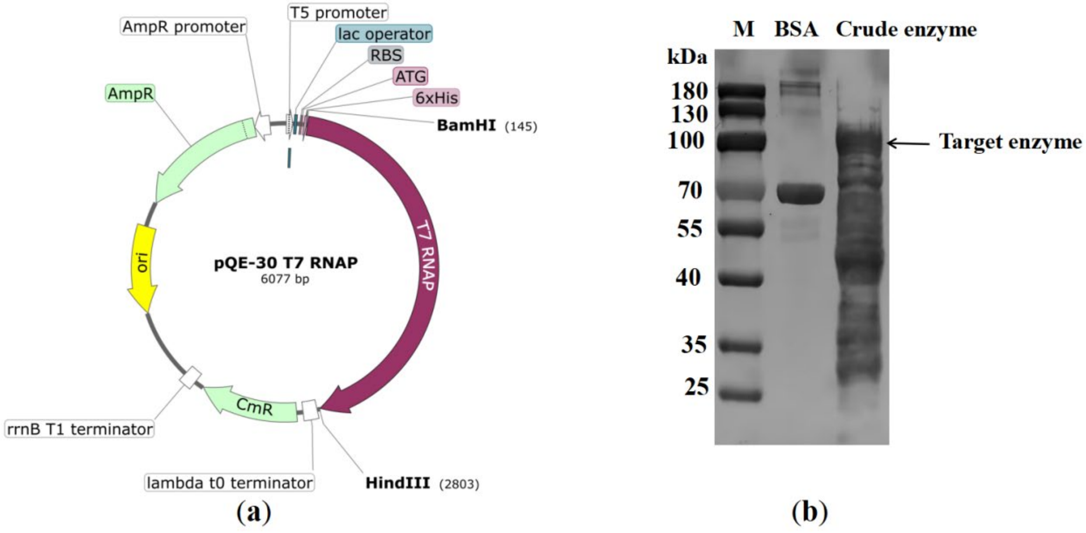

Figure S1. Map of recombinant plasmid pQE-30 and SDS-PAGE analysis of T7 WT.

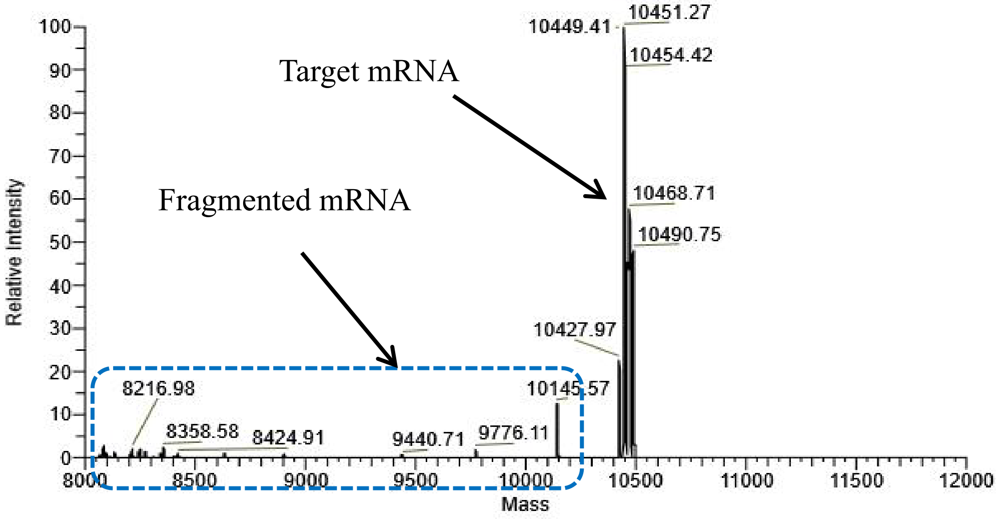

Figure S2. Mass spectra of WT catalyzed mRNA production.

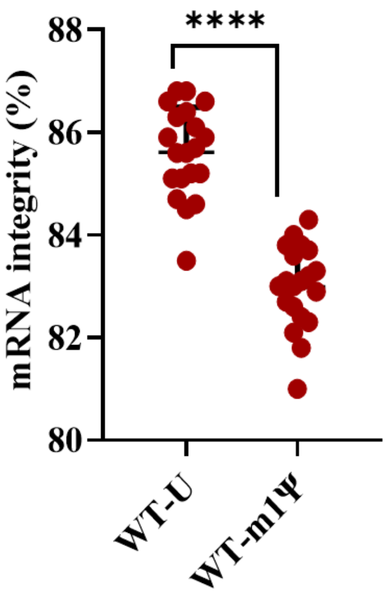

Figure S3. Capillary electrophoresis analysis of mRNA products via use UTP or m1ψTP as substrate (The experiments were replicated 20 times; Error bars represented ±SD; **** p ＜ 0.0001).

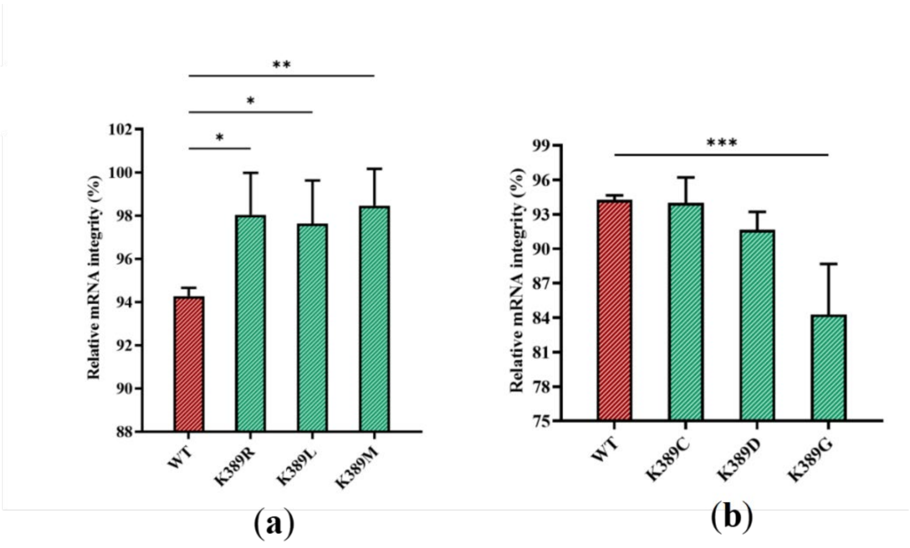

Figure S4. (a) Relative mRNA integrity of higher helix tendency of substitutions (K389A was plotted at 100%); (b) Relative mRNA integrity of lower helix tendency of substitutions (K389A was plotted at 100 %) (The experiments were replicated ≥4 times; error bars represent ±SD; * p ＜ 0.05, ** p ＜ 0.01, *** p ＜ 0.001).

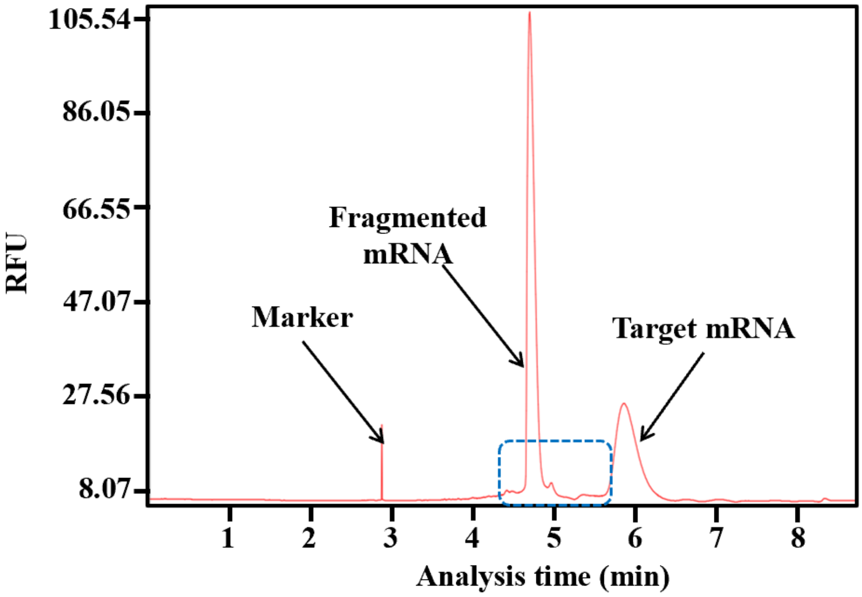

Figure S5. Effect of DNA template sequence inserting Terminator I(rrnBT1) on capillary electrophoresis analysis of mRNA products.

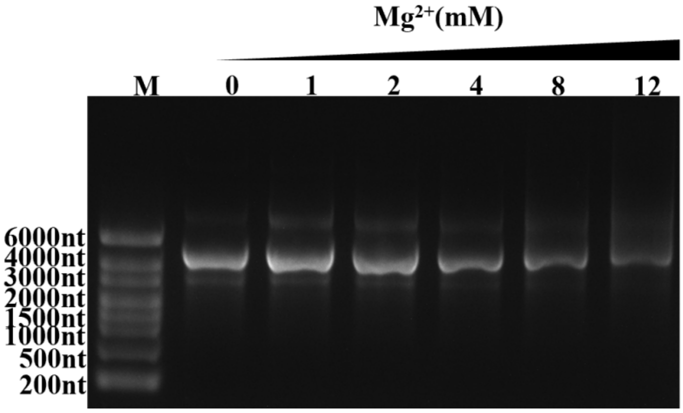

Figure S6. Mg^{2+}-induced degradation of mRNA.

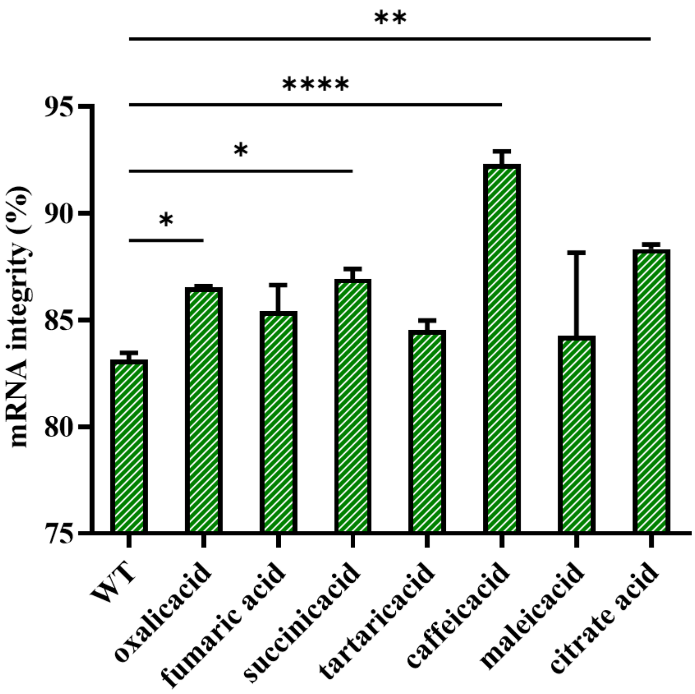

Figure S7. Acids improved relative mRNA integrity (The experiments were replicated≥ 3 times; error bars represent ±SD; * p < 0.05, ** p < 0.01, **** *p* < 0.0001).

Table S1. Optimized DNA template and T7 RNAP K389A improved mRNA integrity (The experiments were replicated ≥ 4 times; error bars represent ±SD).

Substitutions	mRNA Integrity (%)	Yield of mRNA products (μg)
WT-U	85.53±0.99	224.8±6.83
WT-m1Ψ	83.13±0.34	233.7±19.25
K389A-m1Ψ	88.18±1.89	224.7±8.29
K389A-m1Ψ-optimized DNA template	91.55±0.13	222.7±5.60

Table S2. mRNA integrity catalyzed via T7 RNAP substitutions (The experiments were replicated ≥ 4 times; error bars represent ±SD).

Substitutions	mRNA Integrity (%)	Helix propensities (kcal/mol)	Yield of mRNA products (μg)
WT	83.13±0.34	/	233.7±19.25
N171A	85.17±0.78	/	232±16.53
K172A	85.45±1.06	/	226.9±8.72
Q754A	84.95±0.90	/	231.9±7.55
K389A	88.18±1.89	0	224.7±8.29
K389L	86.08±1.77	0.21	227.0±2.34
K389R	86.45±1.71	0.21	234.5±4.60
K389M	86.82±1.50	0.24	228.7±0.70
K389 (WT)	83.13±0.34	0.26	233.7±19.25
K389Q	85.69±1.89	0.39	228.8±1.30
K389E	77.57±2.88	0.40	234.6±3.01
K389I	84.38±1.50	0.41	239.7±5.25
K389W	82.17±1.50	0.49	225.5±2.41
K389S	84.73±1.53	0.50	228.1±1.17
K389Y	81.36±1.96	0.53	228.8±0.57
K389F	84.68±2.66	0.54	236.6±10.38
K389V	79.61±1.57	0.61	228.9±0.42
K389H	85.37±1.54	0.61	237.1±18.51
K389N	85.05±1.52	0.65	228.8±0.68
K389T	84.63±3.47	0.66	238.6±12.16
K389C	82.91±1.94	0.68	221.7±6.05
K389D	80.82±1.39	0.69	231.9±4.00
K389G	74.32±3.88	1	216.2±7.33

Sequences

SEQ NO.1. Optimized cDNA sequence of T7 RNA WT.

ATGAATACAATTAACATAGCTAAAAATGATTTCAGCGATATTGAACTGGCTG
CGATCCCGTTTAATACCTTGGCGGACCATTATGGTGAGCGTCTGGCGCGTGA
GCAACTTGCTCTGGAGCACGAATCCTACGAGATGGGTGAAGCGCGTTTCCG
GAAGATGTTCGAGCGCCAGCTTAAGGCTGGCGAGGTGGCGGATAACGCTG
CTGCGAAGCCGTTGATTACGACCCTGCTGCCGAAAATGATTGCGCGTATTA
ACGATTGGTTCGAGGAAGTGAAAGCAAAGCGTGGTAAACGTCCAACCGCA
TTCCAGTTTCTGCAAGAGATCAAGCCGGAGGCGGTGGCATACATTACGATC
AAAACCACTCTCGCATGTCTCACCAGCGCGGATAATACTACGGTCCAAGCG
GTGGCCAGCGCGATTGGCCGCGCGATTGAGGACGAAGCGCGTTTTGGTCG
CATTCGTGACCTGGAAGCGAAACATTTTAAGAAGAACGTTGAAGAACAAT
TGAATAAACGCGTGGGCCACGTTTACAAGAAAGCGTTCATGCAAGTCGTTG
AAGCTGATATGCTGAGCAAAGGCCTGTTAGGTGGTGAAGCATGGAGCAGC
TGGCATAAGGAGGACTCAATTCACGTCGGCGTGCGGTGCATTGAAATGCTG
ATCGAATCGACCGGTATGGTTAGCCTGCATCGCCAAAACGCCGGCGTGGTG
GGTCAAGATAGCGAAACGATTGAACTGGCGCCAGAGTACGCGGAAGCTAT
CGCTACCAGAGCCGGCGCATTGGCGGGGATCAGCCCGATGTTCCAGCCGTG
TGTTGTACCACCGAAGCCGTGGACCGGTATCACCGGTGGAGGTTACTGGGC
GAATGGTAGACGTCCGTTGGCGTTGGTTCGTACCCATAGCAAAAAGGCTCT
GATGCGTTATGAAGACGTTTACATGCCGGAAGTGTACAAAGCCATCAACAT
CGCGCAGAACACTGCGTGGAAAATCAACAAAAAGGTGTTAGCTGTGGCGA
ACGTAATTACAAAATGGAAGCACTGCCCGGTTGAGGACATCCCGGCTATTG
AACGTGAAGAGCTGCCAATGAAACCAGAGGACATTGACATGAATCCGGAG
GCGCTGACCGCATGGAAACGTGCGGCAGCAGCGGTCTATCGTAAGGATAA
AGCCAGAAAGAGCCGTCGTATCTCCCTGGAATTCATGCTTGAGCAGGCGAA
CAAATTCGCTAACCACAAAGCCATCTGGTTTCCGTATAATATGGATTGGCGT
GGTCGTGTTTACGCGGTATCGATGTTCAACCCGCAGGGTAATGACATGACC
AAGGGTTTGTTGACCTTGGCCAAAGGTAAACCGATTGGCAAAGAAGGTTA
CTATTGGTTGAAGATCCACGGTGCGAACTGCGCAGGGGTGGATAAGGTACC
GTTCCCGGAACGTATTAAATTCATCGAGGAAAACCACGAAAACATTATGGC
GTGCGCAAAGAGCCCGCTGGAAAACACGTGGTGGGCAGAGCAGGACAGT
CCATTCTGCTTCTTGGCGTTCTGTTTCGAATATGCAGGTGTCCAGCACCACG
GCCTGAGCTATAACTGCAGCCTGCCGCTGGCCTTTGATGGTTCTTGTAGCG
GTATTCAACACTTTAGCGCCATGCTGCGCGATGAGGTTGGTGGCAGAGCAG
TGAATCTGCTGCCAAGCGAAACCGTGCAAGATATCTATGGCATTGTGGCTA
AGAAGGTTAATGAAATCCTGCAAGCAGACGCGATTAACGGCACGGACAAC
GAGGTTGTCACCGTGACCGATGAAAACACCGGTGAGATCTCGGAGAAAGT
GAAACTGGGCACCAAAGCCTTGGCTGGCCAATGGCTGGCATACGGCGTTA
CTCGCTCCGTGACCAAGCGTTCGGTGATGACCCTTGCTTACGGCAGCAAAG
AGTTTGGTTTCCGCCAACAGGTTCTGGAGGATACTATCCAACCGGCCATCG
ACTCTGGTAAAGGCCTGATGTTCACGCAGCCGAATCAGGCAGCGGGCTATA
TGGCGAAGCTGATTTGGGAATCGGTTAGCGTTACCGTTGTTGCAGCGGTGG

AGGCTATGAACTGGCTGAAATCTGCGGCGAAGCTGTTAGCTGCGGAGGTG
AAGGACAAGAAGACGGGCGAAATCCTGCGTAAGCGCTGCGCAGTGCATTG
GGTTACGCCGGATGGTTTTCCGGTATGGCAGGAGTACAAAAAGCCTATTCA
GACCCGTCTGAACCTGATGTTTCTGGGTCAATTTCGTCTGCAGCCGACGAT
CAACACCAATAAGGACTCCGAGATCGACGCGCACAAACAGGAGTCTGGCA
TCGCACCCAATTTCGTTCACTCACAGGACGGTTCCCATCTGCGTAAGACCG
TCGTGTGGGCACACGAGAAGTATGGCATTGAGTCGTTTGCATTGATCCACG
ATTCCTTTGGTACCATCCCGGCGGACGCTGCGAACCTGTTTAAAGCGGTTC
GCGAAACCATGGTTGACACCTATGAAAGTTGCGATGTTCTGGCAGACTTTT
ATGATCAGTTCGCCGACCAACTGCATGAGTCCCAGCTGGATAAAATGCCGG
CGTTACCGGCGAAGGGCAACTTAAATCTCCGCGACATCCTGGAATCTGATT
TCGCCTTCGCGTAA

SEQ NO.2. Amino acids sequence of T7 RNAP WT.
MNTINIAKNDFSDIELAAIPFNTLADHYGERLAREQLALEHESYEMGEARFRK
MFERQLKAGEVADNAAAKPLITTLLPKMIARINDWFEEVKAKRGKRPTAFQF
LQEIKPEAVAYITIKTTLACLTSADNTTVQAVASAIGRAIEDEARFGRIRDLEAK
HFKKNVEEQLNKRVGHVYKKAFMQVVEADMLSKGLLGGEAWSSWHKEDSI
HVGVRCIEMLIESTGMVSLHRQNAGVVGQDSETIELAPEYAEAIATRAGALAG
ISPMFQPCVVPPKPWTGITGGGYWANGRRPLALVRTHSKKALMRYEDVYMPE
VYKAINIAQNTAWKINKKVLAVANVITKWKHCPVEDIPAIEREELPMKPEDID
MNPEALTAWKRAAAAVYRKDKARKSRRISLEFMLEQANKFANHKAIWFPYN
MDWRGRVYAVSMFNPQGNDMTKGLLTLAKGKPIGKEGYYWLKIHGANCAG
VDKVPFPERIKFIEENHENIMACAKSPLENTWWAEQDSPFCFLAFCFEYAGVQ
HHGLSYNCSLPLAFDGSCSGIQHFSAMLRDEVGGRAVNLLPSETVQDIYGIVA
KKVNEILQADAINGTDNEVVTVTDENTGEISEKVKLGTKALAGQWLAYGVT
RSVTKRSVMTLAYGSKEFGFRQQVLEDTIQPAIDSGKGLMFTQPNQAAGYMA
KLIWESVSVTVVAAVEAMNWLKSAAKLLAAEVKDKKTGEILRKRCAVHWVT
PDGFPVWQEYKKPIQTRLNLMFLGQFRLQPTINTNKDSEIDAHKQESGIAPNF
VHSQDGSHLRKTVVWAHEKYGIESFALIHDSFGTIPADAANLFKAVRETMVDT
YESCDVLADFYDQFADQLHESQLDKMPALPAKGNLNLRDILESDFAFA

SEQ NO.3. DNA template sequence inserting Terminator I(rrnBT1) sequence.
TAATACGACTCACTATAGGGAGAAAATAACAAATCTCAACACAACATATACA
AAACAAACGAATCTCAAGCAATCAAGCATTCTACTTCTATTGCAGCAATTTA
AATCATTTCTTTTAAAGCAAAAGCAATTTTCTGAAAATTTTCACCATTTACG
AACGATAGCAGCCACCATGTTCGTGTTTCTGGTGCTGCTGCCTCTGGTGAG
CTCCCAGTGCGTGAATCTGACCACAAGGACCCAGCTGCCCCCTGCCTATAC
CAACTCCTTCACACGGGGCGTGTACTATCCCGACAAGGTGTTCCGGAGCAG
CGTGCTGCACTCCACACAGGATCTGTTTCTGCCTTTCTTTTCTAACGTGACC
TGGTTCCACGCCATCCACGTGAGCGGCACCAATGGCACAAAGAGGTTCGA
CAACCCAGTGCTGCCCTTCAATGATGGCGTGTACTTCGCCTCCACCGAGAA
GTCTAATATCATCCGCGGCTGGATCTTTGGCACCACACTGGACAGCAAGAC
ACAGTCCCTGCTGATCGTGAACAATGCCACCAACGTGGTCATCAAGGTGTG

CGAGTTCCAGTTTTGTAACGATCCATTCCTGGGCGTGTACTATCACAAGAAC
AATAAGTCTTGGATGGAGAGCGAGTTTCGCGTGTATTCCTCTGCCAACAAT
TGCACATTTGAGTACGTGTCCCAGCCCTTCCTGATGGACCTGGAGGGCAAG
CAGGGCAATTTCAAGAACCTGCGGGAGTTCGTGTTTAAGAACATCGATGGC
TACTTCAAAATCTACTCCAAGCACACCCCAATCAATCTGGTGAGAGACCTG
CCACAGGGCTTCTCTGCCCTGGAGCCACTGGTGGATCTGCCCATCGGCATC
AACATCACCCGGTTTCAGACACTGCTGGCCCTGCACAGAAGCTACCTGAC
ACCAGGCGACAGCTCCTCTGGATGGACCGCAGGAGCAGCAGCCTACTATG
TGGGCTATCTGCAGCCCAGGACCTTCCTGCTGAAGTACAACGAGAATGGCA
CCATCACAGACGCAGTGGATTGCGCACTGGACCCCCTGTCTGAGACCAAG
TGTACACTGAAGTCCTTTACCGTGGAGAAGGGCATCTATCAGACAAGCAAC
TTCAGGGTGCAGCCTACCGAGTCCATCGTGCGCTTTCCCAATATCACAAAC
CTGTGCCCTTTTGGCGAGGTGTTCAATGCAACCAGGTTCGCAAGCGTGTAC
GCATGGAATAGGAAGCGCATCTCCAACTGCGTGGCCGACTATTCTGTGCTG
TACAATAGCGCCTCCTTCTCTACCTTTAAGTGCTACGGCGTGAGCCCCACAA
AGCTGAATGACCTGTGCTTTACCAACGTGTACGCCGATTCCTTCGTGATCA
GGGGCGACGAGGTGCGCCAGATCGCACCAGGACAGACAGGCAAGATCGC
AGACTACAACTATAAGCTGCCTGACGATTTCACCGGCTGCGTGATCGCCTG
GAACAGCAACAATCTGGATAGCAAAGTGGGCGGCAACTACAATTATCTGTA
CCGGCTGTTTAGAAAGTCTAACCTGAAGCCATTCGAGAGGGACATCTCCAC
AGAAATCTACCAGGCCGGCTCTACCCCCTGCAATGGCGTGGAGGGCTTTAA
CTGTTATTTCCCTCTGCAGAGCTACGGCTTCCAGCCAACAAATGGCGTGGG
CTATCAGCCCTACCGCGTGGTGGTGCTGTCTTTTGAGCTGCTGCACGCCCC
TGCAACAGTGTGCGGACCAAAGAAGTCCACCAATCTGGTGAAGAACAAGT
GCGTGAACTTCAACTTCAACGGACTGACCGGCACAGGCGTGCTGACCGAG
TCCAACAAGAAGTTCCTGCCTTTTCAGCAGTTCGGCAGGGACATCGCAGAT
ACCACAGACGCCGTGCGCGACCCTCAGACCCTGGAGATCCTGGATATCACA
CCATGCTCCTTCGGCGGCGTGTCTGTGATCACACCAGGCACCAATACAAGC
AACCAGGTGGCCGTGCTGTATCAGGACGTGAACTGTACCGAGGTGCCCGT
GGCAATCCACGCAGATCAGCTGACCCCTACATGGCGGGTGGGCATCAAATA
AAACGAAAGGCTCAGTCGAAAGACTGGGCCTTTTCGTTTTATCTGTTGTTT
GTCTACTCTACCGGCAGCAACGTGTTCCAGACAAGAGCCGGATGCCTGATC
GGAGCAGAGCACGTGAACAATAGCTATGAGTGCGACATCCCTATCGGCGCC
GGCATCTGTGCCTCCTACCAGACCCAGACAAACTCCCCAAGGAGAGCACG
GTCTGTGGCAAGCCAGTCCATCATCGCCTATACCATGAGCCTGGGCGCCGA
GAACTCCGTGGCCTACTCCAACAATTCTATCGCCATCCCTACCAATTTCACA
ATCTCCGTGACCACAGAGATCCTGCCAGTGAGCATGACCAAGACATCCGTG
GACTGCACAATGTATATCTGTGGCGATTCCACCGAGTGCTCTAATCTGCTGC
TGCAGTACGGCTCTTTTTGTACCCAGCTGAACAGAGCCCTGACAGGCATCG
CCGTGGAGCAGGACAAGAATACACAGGAGGTGTTCGCCCAGGTGAAGCA
AATCTACAAGACCCCACCCATCAAGGACTTTGGCGGCTTCAACTTTAGCCA
GATCCTGCCCGATCCTAGCAAGCCATCCAAGCGGTCTTTTATCGAGGACCT
GCTGTTCAATAAGGTGACCCTGGCCGATGCCGGCTTCATCAAGCAGTATGG
CGATTGCCTGGGCGACATCGCCGCCAGAGACCTGATCTGTGCCCAGAAGTT

TAACGGCCTGACCGTGCTGCCTCCACTGCTGACAGATGAGATGATCGCCCA
GTACACATCTGCCCTGCTGGCAGGCACCATCACAAGCGGATGGACCTTCGG
CGCAGGAGCCGCCCTGCAGATCCCCTTTGCCATGCAGATGGCCTATCGGTT
CAATGGCATCGGCGTGACCCAGAATGTGCTGTACGAGAACCAGAAGCTGA
TCGCCAATCAGTTTAACTCCGCCATCGGCAAGATCCAGGACTCTCTGAGCT
CCACAGCAAGCGCCCTGGGCAAGCTGCAGGATGTGGTGAATCAGAACGCC
CAGGCCCTGAACACCCTGGTGAAGCAGCTGTCTAGCAATTTCGGCGCCATC
TCCTCTGTGCTGAACGATATCCTGAGCCGGCTGGACAAGGTGGAGGCAGA
GGTGCAGATCGACCGGCTGATCACAGGCAGACTGCAGTCCCTGCAGACCT
ACGTGACACAGCAGCTGATCAgGGCAGCAGAGATCAGGGCATCTGCCAAC
CTGGCAGCAACCAAGATGAGCGAGTGCGTGCTGGGCCAGTCCAAGAGAGT
GGACTTTTGTGGCAAGGGCTATCACCTGATGAGCTTCCCACAGTCCGCCCC
TCACGGAGTGGTGTTTCTGCACGTGACCTACGTGCCAGCCCAGGAGAAGA
ACTTCACCACAGCACCAGCAATCTGCCACGATGGCAAGGCACACTTTCCTA
GGGAGGGCGTGTTCGTGAGCAATGGCACCCACTGGTTTGTGACACAGCGC
AACTTCTACGAGCCACAGATCATCACCACAGACAATACATTCGTGTCCGGC
AACTGTGACGTGGTCATCGGCATCGTGAACAATACCGTGTATGATCCTCTGC
AGCCAGAGCTGGACTCTTTTAAGGAGGAGCTGGATAAGTACTTCAAGAAC
CACACCAGCCCCGACGTGGATCTGGGCGACATCTCTGGCATCAATGCCAGC
GTGGTGAACATCCAGAAGGAGATCGACAGGCTGAATGAGGTGGCCAAGAA
TCTGAACGAGTCCCTGATCGATCTGCAGGAGCTGGGCAAGTATGAGCAGTA
CATCAAGTGGCCCTGGTATATCTGGCTGGGCTTCATCGCCGGCCTGATCGCC
ATCGTGATGGTGACCATCATGCTGTGCTGTATGACAAGCTGCTGTTCCTGCC
TGAAGGGCTGCTGTTCTTGTGGCAGCTGCTGTAAGTTTGATGAGGACGATA
GCGAGCCTGTGCTGAAGGGCGTGAAGCTGCACTACACCTGAGAATTCAAC
CAGCCTCAAGAACACCCGAATGGAGTCTCTAAGCTACATAATACCAACTTA
CACTTTACAAAATGTTGTCCCCCAAAATGTAGCCATTCGTATCTGCTCCTAA
TAAAAAGAAAGTTTCTTCACATTCTAACCAGCCTCAAGAACACCCGAATGG
AGTCTCTAAGCTACATAATACCAACTTACACTTTACAAAATGTTGTCCCCCA
AAATGTAGCCATTCGTATCTGCTCCTAATAAAAAGAAAGTTTCTTCACATTC
TAAGCTTAAAAAAAAAAAAAAAAAAAAAAAAAAAAAAAAAAAAAAAAAAAA
AAAAAAAAAAAAAAAAAAAAAAAAAAAAAAAAAAAAAAAAAAAAAAAAAA
AAAAAAAAAAAAAAAAAAAAAAAAAAAAAAAAA

SEQ NO.4. Original DNA template sequence.
TAATACGACTCACTATAGGGAGAAAATAACAAATCTCAACACAACATATACA
AAACAAACGAATCTCAAGCAATCAAGCATTCTACTTCTATTGCAGCAATTTA
AATCATTTCTTTTAAAGCAAAAGCAATTTTCTGAAAATTTTCACCATTTACG
AACGATAGCAGCCACCATGTTCGTGTTTCTGGTGCTGCTGCCTCTGGTGAG
CTCCCAGTGCGTGAATCTGACCACAAGGACCCAGCTGCCCCCTGCCTATAC
CAACTCCTTCACACGGGGCGTGTACTATCCCGACAAGGTGTTCCGGAGCAG
CGTGCTGCACTCCACACAGGATCTGTTTCTGCCTTTCTTTTCTAACGTGACC
TGGTTCCACGCCATCCACGTGAGCGGCACCAATGGCACAAAGAGGTTCGA
CAACCCAGTGCTGCCCTTCAATGATGGCGTGTACTTCGCCTCCACCGAGAA

GTCTAATATCATCCGCGGCTGGATCTTTGGCACCACACTGGACAGCAAGAC
ACAGTCCCTGCTGATCGTGAACAATGCCACCAACGTGGTCATCAAGGTGTG
CGAGTTCCAGTTTTGTAACGATCCATTCCTGGGCGTGTACTATCACAAGAAC
AATAAGTCTTGGATGGAGAGCGAGTTTCGCGTGTATTCCTCTGCCAACAAT
TGCACATTTGAGTACGTGTCCCAGCCCTTCCTGATGGACCTGGAGGGCAAG
CAGGGCAATTTCAAGAACCTGCGGGAGTTCGTGTTTAAGAACATCGATGGC
TACTTCAAAATCTACTCCAAGCACACCCCAATCAATCTGGTGAGAGACCTG
CCACAGGGCTTCTCTGCCCTGGAGCCACTGGTGGATCTGCCCATCGGCATC
AACATCACCCGGTTTCAGACACTGCTGGCCCTGCACAGAAGCTACCTGAC
ACCAGGCGACAGCTCCTCTGGATGGACCGCAGGAGCAGCAGCCTACTATG
TGGGCTATCTGCAGCCCAGGACCTTCCTGCTGAAGTACAACGAGAATGGCA
CCATCACAGACGCAGTGGATTGCGCACTGGACCCCCTGTCTGAGACCAAG
TGTACACTGAAGTCCTTTACCGTGGAGAAGGGCATCTATCAGACAAGCAAC
TTCAGGGTGCAGCCTACCGAGTCCATCGTGCGCTTTCCCAATATCACAAAC
CTGTGCCCTTTTGGCGAGGTGTTCAATGCAACCAGGTTCGCAAGCGTGTAC
GCATGGAATAGGAAGCGCATCTCCAACTGCGTGGCCGACTATTCTGTGCTG
TACAATAGCGCCTCCTTCTCTACCTTTAAGTGCTACGGCGTGAGCCCCACAA
AGCTGAATGACCTGTGCTTTACCAACGTGTACGCCGATTCCTTCGTGATCA
GGGGCGACGAGGTGCGCCAGATCGCACCAGGACAGACAGGCAAGATCGC
AGACTACAACTATAAGCTGCCTGACGATTTCACCGGCTGCGTGATCGCCTG
GAACAGCAACAATCTGGATAGCAAAGTGGGCGGCAACTACAATTATCTGTA
CCGGCTGTTTAGAAAGTCTAACCTGAAGCCATTCGAGAGGGACATCTCCAC
AGAAATCTACCAGGCCGGCTCTACCCCCTGCAATGGCGTGGAGGGCTTTAA
CTGTTATTTCCCTCTGCAGAGCTACGGCTTCCAGCCAACAAATGGCGTGGG
CTATCAGCCCTACCGCGTGGTGGTGCTGTCTTTTGAGCTGCTGCACGCCCC
TGCAACAGTGTGCGGACCAAAGAAGTCCACCAATCTGGTGAAGAACAAGT
GCGTGAACTTCAACTTCAACGGACTGACCGGCACAGGCGTGCTGACCGAG
TCCAACAAGAAGTTCCTGCCTTTTCAGCAGTTCGGCAGGGACATCGCAGAT
ACCACAGACGCCGTGCGCGACCCTCAGACCCTGGAGATCCTGGATATCACA
CCATGCTCCTTCGGCGGCGTGTCTGTGATCACACCAGGCACCAATACAAGC
AACCAGGTGGCCGTGCTGTATCAGGACGTGAACTGTACCGAGGTGCCCGT
GGCAATCCACGCAGATCAGCTGACCCCTACATGGCGGGTGTACTCTACCGG
CAGCAACGTGTTCCAGACAAGAGCCGGATGCCTGATCGGAGCAGAGCACG
TGAACAATAGCTATGAGTGCGACATCCCTATCGGCGCCGGCATCTGTGCCTC
CTACCAGACCCAGACAAACTCCCCAAGGAGAGCACGGTCTGTGGCAAGCC
AGTCCATCATCGCCTATACCATGAGCCTGGGCGCCGAGAACTCCGTGGCCT
ACTCCAACAATTCTATCGCCATCCCTACCAATTTCACAATCTCCGTGACCAC
AGAGATCCTGCCAGTGAGCATGACCAAGACATCCGTGGACTGCACAATGTA
TATCTGTGGCGATTCCACCGAGTGCTCTAATCTGCTGCTGCAGTACGGCTCT
TTTTGTACCCAGCTGAACAGAGCCCTGACAGGCATCGCCGTGGAGCAGGA
CAAGAATACACAGGAGGTGTTCGCCCAGGTGAAGCAAATCTACAAGACCC
CACCCATCAAGGACTTTGGCGGCTTCAACTTTAGCCAGATCCTGCCCGATC
CTAGCAAGCCATCCAAGCGGTCTTTTATCGAGGACCTGCTGTTCAATAAGG
TGACCCTGGCCGATGCCGGCTTCATCAAGCAGTATGGCGATTGCCTGGGCG

ACATCGCCGCCAGAGACCTGATCTGTGCCCAGAAGTTTAACGGCCTGACCG
TGCTGCCTCCACTGCTGACAGATGAGATGATCGCCCAGTACACATCTGCCC
TGCTGGCAGGCACCATCACAAGCGGATGGACCTTCGGCGCAGGAGCCGCC
CTGCAGATCCCCTTTGCCATGCAGATGGCCTATCGGTTCAATGGCATCGGCG
TGACCCAGAATGTGCTGTACGAGAACCAGAAGCTGATCGCCAATCAGTTTA
ACTCCGCCATCGGCAAGATCCAGGACTCTCTGAGCTCCACAGCAAGCGCC
CTGGGCAAGCTGCAGGATGTGGTGAATCAGAACGCCCAGGCCCTGAACAC
CCTGGTGAAGCAGCTGTCTAGCAATTTCGGCGCCATCTCCTCTGTGCTGAA
CGATATCCTGAGCCGGCTGGACAAGGTGGAGGCAGAGGTGCAGATCGACC
GGCTGATCACAGGCAGACTGCAGTCCCTGCAGACCTACGTGACACAGCAG
CTGATCAgGGCAGCAGAGATCAGGGCATCTGCCAACCTGGCAGCAACCAA
GATGAGCGAGTGCGTGCTGGGCCAGTCCAAGAGAGTGGACTTTTGTGGCA
AGGGCTATCACCTGATGAGCTTCCCACAGTCCGCCCCTCACGGAGTGGTGT
TTCTGCACGTGACCTACGTGCCAGCCCAGGAGAAGAACTTCACCACAGCA
CCAGCAATCTGCCACGATGGCAAGGCACACTTTCCTAGGGAGGGCGTGTTC
GTGAGCAATGGCACCCACTGGTTTGTGACACAGCGCAACTTCTACGAGCC
ACAGATCATCACCACAGACAATACATTCGTGTCCGGCAACTGTGACGTGGT
CATCGGCATCGTGAACAATACCGTGTATGATCCTCTGCAGCCAGAGCTGGA
CTCTTTTAAGGAGGAGCTGGATAAGTACTTCAAGAACCACACCAGCCCCGA
CGTGGATCTGGGCGACATCTCTGGCATCAATGCCAGCGTGGTGAACATCCA
GAAGGAGATCGACAGGCTGAATGAGGTGGCCAAGAATCTGAACGAGTCCC
TGATCGATCTGCAGGAGCTGGGCAAGTATGAGCAGTACATCAAGTGGCCCT
GGTATATCTGGCTGGGCTTCATCGCCGGCCTGATCGCCATCGTGATGGTGAC
CATCATGCTGTGCTGTATGACAAGCTGCTGTTCCTGCCTGAAGGGCTGCTG
TTCTTGTGGCAGCTGCTGTAAGTTTGATGAGGACGATAGCGAGCCTGTGCT
GAAGGGCGTGAAGCTGCACTACACCTGAGAATTCAACCAGCCTCAAGAAC
ACCCGAATGGAGTCTCTAAGCTACATAATACCAACTTACACTTTACAAAATG
TTGTCCCCCAAAATGTAGCCATTCGTATCTGCTCCTAATAAAAAGAAAGTTT
CTTCACATTCTAACCAGCCTCAAGAACACCCGAATGGAGTCTCTAAGCTAC
ATAATACCAACTTACACTTTACAAAATGTTGTCCCCCAAAATGTAGCCATTC
GTATCTGCTCCTAATAAAAAGAAAGTTTCTTCACATTCTAAGCTTAAAAAAA
AAAAAAAAAAAAAAAAAAAAAAAAAAAAAAAAAAAAAAAAAAAAAAA
AAAAAAAAAAAAAAAAAAAAAAAAAAAAAAAAAAAAAAAAAAAAAAA
AAAAAAAAAAAAAAAAAAA

SEQ NO.5. Optimized DNA template sequence of Moderna mRNA-1273 vaccine.
TAATACGACTCACTATAGGGAAATAAGAGAGAAAAGAAGAGTAAGAAGAA
ATATAAGACCCCGGCGCCGCCACCATGTTCGTGTTCCTGGTGCTGCTGCCC
CTGGTGAGCAGCCAGTGCGTGAACCTGACCACCCGGACCCAGCTGCCACC
AGCCTACACCAACAGCTTCACCCGGGGCGTCTACTACCCCGACAAGGTGTT
CCGGAGCAGCGTCCTGCACAGCACCCAGGACCTGTTCCTGCCCTTCTTCAG
CAACGTGACCTGGTTCCACGCCATCCACGTGAGCGGCACCAACGGCACCA
AGCGGTTCGACAACCCCGTGCTGCCCTTCAACGACGGCGTGTACTTCGCCA

GCACCGAGAAGAGCAACATCATCCGGGGCTGGATCTTCGGCACCACCCTG
GACAGCAAGACCCAGAGCCTGCTGATCGTGAATAACGCCACCAACGTGGT
GATCAAGGTGTGCGAGTTCCAGTTCTGCAACGACCCCTTCCTGGGCGTGTA
CTACCACAAGAACAACAAGAGCTGGATGGAGAGCGAGTTCCGGGTGTACA
GCAGCGCCAACAACTGCACCTTCGAGTACGTGAGCCAGCCCTTCCTGATG
GACCTGGAGGGCAAGCAGGGCAACTTCAAGAACCTGCGGGAGTTCGTGTT
CAAGAACATCGACGGCTACTTCAAGATCTACAGCAAGCACACCCCAATCA
ACCTGGTGCGGGATCTGCCCCAGGGCTTCTCAGCCCTGGAGCCCCTGGTG
GACCTGCCCATCGGCATCAACATCACCCGGTTCCAGACCCTGCTGGCCCTG
CACCGGAGCTACCTGACCCCAGGCGACAGCAGCAGCGGGTGGACAGCAG
GCGCGGCTGCTTACTACGTGGGCTACCTGCAGCCCCGGACCTTCCTGCTGA
AGTACAACGAGAACGGCACCATCACCGACGCCGTGGACTGCGCCCTGGAC
CCTCTGAGCGAGACCAAGTGCACCCTGAAGAGCTTCACCGTGGAGAAGGG
CATCTACCAGACCAGCAACTTCCGGGTGCAGCCCACCGAGAGCATCGTGC
GGTTCCCCAACATCACCAACCTGTGCCCCTTCGGCGAGGTGTTCAACGCCA
CCCGGTTCGCCAGCGTGTACGCCTGGAACCGGAAGCGGATCAGCAACTGC
GTGGCCGACTACAGCGTGCTGTACAACAGCGCCAGCTTCAGCACCTTCAA
GTGCTACGGCGTGAGCCCCACCAAGCTGAACGACCTGTGCTTCACCAACG
TGTACGCCGACAGCTTCGTGATCCGTGGCGACGAGGTGCGGCAGATCGCA
CCCGGCCAGACAGGCAAGATCGCCGACTACAACTACAAGCTGCCCGACGA
CTTCACCGGCTGCGTGATCGCCTGGAACAGCAACAACCTCGACAGCAAGG
TGGGCGGCAACTACAACTACCTGTACCGGCTGTTCCGGAAGAGCAACCTG
AAGCCCTTCGAGCGGGACATCAGCACCGAGATCTACCAAGCCGGCTCCAC
CCCTTGCAACGGCGTGGAGGGCTTCAACTGCTACTTCCCTCTGCAGAGCTA
CGGCTTCCAGCCCACCAACGGCGTGGGCTACCAGCCCTACCGGGTGGTGG
TGCTGAGCTTCGAGCTGCTGCACGCCCCAGCCACCGTGTGTGGCCCCAAG
AAGAGCACCAACCTGGTGAAGAACAAGTGCGTGAACTTCAACTTCAACGG
CCTTACCGGCACCGGCGTGCTGACCGAGAGCAACAAGAAATTCCTGCCCT
TTCAGCAGTTCGGCCGGGACATCGCCGACACCACCGACGCTGTGCGGGAT
CCCCAGACCCTGGAGATCCTGGACATCACCCCTTGCAGCTTCGGCGGCGTG
AGCGTGATCACCCCAGGCACCAACACCAGCAACCAGGTGGCCGTGCTGTA
CCAGGACGTGAACTGCACCGAGGTGCCCGTGGCCATCCACGCCGACCAGC
TGACACCCACCTGGCGGGTCTACAGCACCGGCAGCAACGTGTTCCAGACC
CGGGCCGGTTGCCTGATCGGCGCCGAGCACGTGAACAACAGCTACGAGTG
CGACATCCCCATCGGCGCCGGCATCTGTGCCAGCTACCAGACCCAGACCAA
TTCACCCCGGAGGGCAAGGAGCGTGGCCAGCCAGAGCATCATCGCCTACA
CCATGAGCCTGGGCGCCGAGAACAGCGTGGCCTACAGCAACAACAGCATC
GCCATCCCCACCAACTTCACCATCAGCGTGACCACCGAGATTCTGCCCGTG
AGCATGACCAAGACCAGCGTGGACTGCACCATGTACATCTGCGGCGACAG
CACCGAGTGCAGCAACCTGCTGCTGCAGTACGGCAGCTTCTGCACCCAGC
TGAACCGGGCCCTGACCGGCATCGCCGTGGAGCAGGACAAGAACACCCA
GGAGGTGTTCGCCCAGGTGAAGCAGATCTACAAGACCCCTCCCATCAAGG
ACTTCGGCGGCTTCAACTTCAGCCAGATCCTGCCCGACCCCAGCAAGCCC
AGCAAGCGGAGCTTCATCGAGGACCTGCTGTTCAACAAGGTGACCCTAGC

CGACGCCGGCTTCATCAAGCAGTACGGCGACTGCCTCGGCGACATAGCCG
CCCGGGACCTGATCTGCGCCCAGAAGTTCAACGGCCTGACCGTGCTGCCT
CCCCTGCTGACCGACGAGATGATCGCCCAGTACACCAGCGCCCTGTTAGCC
GGAACCATCACCAGCGGCTGGACTTTCGGCGCTGGAGCCGCTCTGCAGAT
CCCCTTCGCCATGCAGATGGCCTACCGGTTCAACGGCATCGGCGTGACCCA
GAACGTGCTGTACGAGAACCAGAAGCTGATCGCCAACCAGTTCAACAGCG
CCATCGGCAAGATCCAGGACAGCCTGAGCAGCACCGCTAGCGCCCTGGGC
AAGCTGCAGGACGTGGTGAACCAGAACGCCCAGGCCCTGAACACCCTGGT
GAAGCAGCTGAGCAGCAACTTCGGCGCCATCAGCAGCGTGCTGAACGACA
TCCTGAGCCGGCTGGACCCTCCCGAGGCCGAGGTGCAGATCGACCGGCTG
ATCACTGGCCGGCTGCAGAGCCTGCAGACCTACGTGACCCAGCAGCTGAT
CCGGGCCGCCGAGATTCGGGCCAGCGCCAACCTGGCCGCCACCAAGATGA
GCGAGTGCGTGCTGGGCCAGAGCAAGCGGGTGGACTTCTGCGGCAAGGG
CTACCACCTGATGAGCTTTCCCCAGAGCGCACCCCACGGAGTGGTGTTCCT
GCACGTGACCTACGTGCCCGCCCAGGAGAAGAACTTCACCACCGCCCCAG
CCATCTGCCACGACGGCAAGGCCCACTTTCCCCGGGAGGGCGTGTTCGTG
AGCAACGGCACCCACTGGTTCGTGACCCAGCGGAACTTCTACGAGCCCCA
GATCATCACCACCGACAACACCTTCGTGAGCGGCAACTGCGACGTGGTGA
TCGGCATCGTGAACAACACCGTGTACGATCCCCTGCAGCCCGAGCTGGAC
AGCTTCAAGGAGGAGCTGGACAAGTACTTCAAGAATCACACCAGCCCCGA
CGTGGACCTGGGCGACATCAGCGGCATCAACGCCAGCGTGGTGAACATCC
AGAAGGAGATCGATCGGCTGAACGAGGTGGCCAAGAACCTGAACGAGAG
CCTGATCGACCTGCAGGAGCTGGGCAAGTACGAGCAGTACATCAAGTGGC
CCTGGTACATCTGGCTGGGCTTCATCGCCGGCCTGATCGCCATCGTGATGGT
GACCATCATGCTGTGCTGCATGACCAGCTGCTGCAGCTGCCTGAAGGGCTG
TTGCAGCTGCGGCAGCTGCTGCAAGTTCGACGAGGACGACAGCGAGCCCG
TGCTGAAGGGCGTGAAGCTGCACTACACCTGATAATAGGCTGGAGCCTCGG
TGGCCTAGCTTCTTGCCCCTTGGGCCTCCCCCCAGCCCCTCCTCCCCTTCCT
GCACCCGTACCCCCGTGGTCTTTGAATAAAGTCTGAGTGGGCGGCAAAAA
AAAAAAAAAAAAAAAAAAAAAAAAAAAAAAAAAAAAAAAAAAAAAAAAAA
AAAAAAAAAAAAAAAAAAAAAAAAAAAAAAAAAAAAAAAAAAAAAAAAAA
AAAAAAAAAAAAAAAAAAAAAA

SEQ NO.6. 30nt.
CAGTAATACGACTCACTATAgatgatgatgatgatgatgatgatgatgat
